# Development of mental health first-aid guidelines for a person after a potentially traumatic event: A Delphi expert consensus study in Argentina and Chile

**DOI:** 10.1186/s12888-024-05631-4

**Published:** 2024-04-17

**Authors:** Martín Agrest, Thamara Tapia-Muñoz, Esteban Encina-Zúñiga, Isidora Vidal-Zamora, Sara Ardila-Gómez, Rubén Alvarado, Eduardo A Leiderman, Nicola Reavley

**Affiliations:** 1 Proyecto Suma. Güemes 4130 (1425), Ciudad Autónoma de Buenos Aires, Buenos Aires, Argentina; 2 Facultad de Psicología, Instituto de Investigaciones, Universidad de Buenos Aires, Buenos Aires, Argentina; 3 Department of Behavioural Science and Health, University College London, London, UK; 4 School of Public Health, Faculty of Medicine, Universidad de Chile, Santiago, Chile; 5 Department of Psychology, Faculty of Social Sciences, Universidad de Chile, Santiago, Chile; 6 Consejo Nacional de Investigaciones Científicas y Técnicas (CONICET), Buenos Aires, Argentina; 7 Department of Public Health, School of Medicine, Faculty of Medicine, Universidad de Valparaíso, Valparaíso, Chile; 8 Departamento de Neurociencias, Facultad de Ciencias Sociales, Universidad de Palermo, Buenos Aires, Argentina; 9 Centre for Mental Health, Melbourne School of Population and Global Health, University of Melbourne, Victoria, Australia

**Keywords:** Trauma, Mental health first aid (MHFA), Cultural adaptation, Delphi study, Chile, Argentina

## Abstract

**Background:**

Exposure to potentially traumatic events increases the risk of a person developing a mental disorder. Training community members to offer support to a person during and after a traumatic situation may help lower this risk. This study reports on the cultural adaptation of Australian mental health first aid guidelines for individuals exposed to a potentially traumatic event to the Chilean and Argentinian context.

**Methods:**

A Delphi expert consensus study was conducted with two panels of experts, one of people with lived experience of trauma (either their own or as a carer; *n* = 26) and another one of health professionals (*n* = 41). A total of 158 items, drawn from guidelines developed by Australian experts in 2019, were translated to Spanish and evaluated in a two-round survey process. The panellists were asked to rate each item on a five-point Likert scale; statements were included in the final guidelines if 80% of both panels endorsed the item as “essential” or “important”.

**Results:**

Consensus was achieved on 142 statements over two survey rounds. A total of 102 statements were included from the English-language guidelines, and 40 locally generated statements were accepted in the second round. Local experts endorsed a larger number of items compared to their counterparts in Australia and emphasised the importance of acknowledging the first aider’s limitations, both personally and as part of their helping role. Additional items about working as a team with other first responders and considering helping the person’s significant others were endorsed by the local panellists.

**Conclusions:**

The study showed a high level of acceptance of the original actions suggested for inclusion in the guidelines for Australia, but also a significant number of new statements that highlight the importance of the adaptation process. Further research on the dissemination of these guidelines into a Mental Health First Aid training course for Chile and Argentina is still required.

**Supplementary Information:**

The online version contains supplementary material available at 10.1186/s12888-024-05631-4.

## Introduction

Latin America and the Caribbean, the world’s most economically unequal and the second-most disaster-prone region [1], sadly offers exposure to a large range of potentially traumatic events (PTEs; defined by the DSM-5 [2] as “actual or threatened death, severe injury, or sexual violence”) for its 671 million inhabitants [3]. Natural or human-caused disasters and catastrophes (like earthquakes, fire inside populated buildings, extreme political violence, terrorist attacks, forced migration, and wars), and individual incidents (being robbed, kidnapped or attacked), frequently challenge its citizens, with climate breakdown a powerful catalyst [4]. According to a worldwide study [5], which used the broader DSM-IV definition of trauma [6], five types of traumatic events accounted for over half of all exposures (i.e., witnessing death or serious injury, the unexpected death of a loved one, being mugged, being in a life-threatening automobile accident, and experiencing a life-threatening illness or injury). Exposure varied by country, and sociodemographic characteristics, history of prior traumatic events and interpersonal violence had the strongest associations with subsequent traumatic events. Collective violence, relatively common in Latin America, ranked low as a risk factor for trauma. Importantly, scholars in the region have coined and developed the concept of “psychosocial trauma” [7–10] to make sense of collective violence and its psychosocial and human rights consequences (including intergroup hostility, social polarisation, the destruction of fundamental beliefs, and family and community destruction) [11], highlighting the need to focus on this underestimated source of trauma in these countries.

It has been estimated that one-third of individuals exposed to a traumatic event may develop post-traumatic stress disorder (PTSD) or other disorders, including anxiety, depression, and acute stress disorder [12, 13], although estimates depend on the type of trauma and several other factors that may have a mediating role [14]. According to an Israeli study, terrorist attack survivors developed PTSD at twice the rate of survivors of motor vehicle accidents (37.8% versus 18.7%) [15], highlighting that some PTEs may be more likely than others to lead to mental disorders. Other studies showed even greater differences, ranging from 15 to 75% of individuals developing PTSD following different PTEs [16].

Furthermore, direct exposure to a PTE (or indirectly, as a witness) may occur in over two thirds of the population [5] and according to some studies it could be as high as 90% [12, 17]. Some people may experience multiple traumatic events [18]. Countries exposed to natural disasters (e.g., Chile), developing countries and other countries with high levels of inequality (e.g., Chile and Argentina) may combine frequent PTE exposure with chaotic urbanisation and underprepared or underfinanced response teams, resulting in an explosive combination and a higher risk [19]. Furthermore, Chile and Argentina share a history of state and political violence that has been transmitted across generations [20] and has shaped cultural processes that included the elaboration of collective trauma, social mourning, and calls for truth and justice in both countries [21].

Scars may not always be visible and, despite mental health being as important as physical health, psychological consequences of PTEs have often been overlooked [22]. More recently, there have been growing calls for interventions to buffer the impacts of PTEs [23]. Resiliency theory may provide a framework for understanding why some individuals develop a mental disorder after a PTE while others do not [24, 25]. Fergus and Zimmerman [26] described three general classes of resilience models–protective, compensatory, and challenge– which affect how to tailor support provided to someone exposed to a PTE. In particular, the protective model of resilience refers to a process in which a positive factor may buffer or moderate the negative effect of a risk factor on an outcome [27, 28]. Availability of adequate protective factors may thus become crucial when organising community help surrounding the exposure to PTEs. Social support is one of the factors that can have such a protective role, especially when the social network reacts in a positive way to traumatic situations that are “visually distressing, unambiguous, collectively shared and may even attribute heroic characteristics to the victims” [14, 29]. However, despite potentially benefiting from support from the social network, other PTEs may not elicit such positive feelings and require that people in the social network of a person exposed to a PTE receive further mental health training to provide adequate support. Adding to this, COVID-19, an unprecedented, generalised PTE that hit Latin America and the Caribbean particularly hard (despite comprising only 8.4% of the global population it accounted for 30.3% of all deaths [1]), accelerated the awareness of a rising need for appropriate support for individuals exposed to PTEs.

### Psychological first aid response to potentially traumatic events

Initial strategic responses to PTEs have not been uncommon across the world. During World War II, Psychological First Aid (PFA) was introduced for the personnel of the Merchant Marine, and was used in war and in peace [30]. PFA is an evidence-informed modular approach to help children, adolescents, adults, and families in the immediate aftermath of disaster and terrorism. It was originally designed to be delivered by mental health and other disaster response workers and spread to volunteers and the general public. PFA aims to reduce the initial distress caused by a traumatic event and to foster short- and long-term adaptive functioning and coping [31]. PFA is widespread [32] and has consistently been recommended as an early intervention for disaster survivors and in international treatment guidelines for PTSD [33]. The World Health Organization (WHO) adopted a form of PFA and translated this initiative into 20 different languages [34]. Its implementation has included training of different lengths (ranging from a single session to multiple sessions), providers (e.g., mental health professionals and others with nonspecific backgrounds facilitated the intervention), and settings (individually and in groups). Basic principles of PFA are: offering non-intrusive practical care and support, helping people to address basic needs (e.g., food and water), listening without pressuring people to talk, comforting people and helping them to feel calm, helping people connect to information, services and social supports, and protecting people from further harm [35].

However, while PFA is not primarily intended (although suitable) for people from the community that may occasionally come into contact with (and be able to help) people who have experienced a PTE, it is largely focused on support in the immediate aftermath of an event. Other programs such as Mental Health First Aid, which cover a broader range of mental health problems and crises are targeted to non-professional helpers and may be better suited to providing support weeks and months after potentially traumatic events, that are also applicable to responding to traumatic experiences.

### Chilean and Argentinian first response system to traumatic events

Throughout its history Chile has been particularly exposed to natural disasters like earthquakes, volcanic activity, and tsunamis, along with other threats generated by human action. In May 1960, the world’s most powerful ever recorded earthquake struck the town of Valdivia in southern Chile and became known as the “Great Chilean Earthquake”, killing 6,000 people in Chile and–due to the tsunami– also 130 in Japan and 60 in Hawaii. By 2008, the Chilean Minister of Health incorporated a mental health component to the National Plan for Emergencies and Disasters which resulted in many officers trained in management of resources and interventions for the protection of mental health in connection to disasters. However, in February 2010, an 8.8 Richter scale earthquake that affected 80% of the Chilean population causing 524 deaths and 31 missing persons [36], showed significant deficiencies at the response system level that included a limited awareness and participation by community members [37]. In 2015, several simultaneous catastrophic events impacted Chile (including earthquakes, tsunamis, volcanos, flooding rain, and alluvium) and catalysed intersectoral and collaborative work with the Government of Japan and the Japanese International Cooperation Agency (JICA), which also aligned with up-to-date scientific literature, guidelines and recommendations from international agencies, and learnings from previous natural disasters in Chile. This consortium gave way in 2019 to a Mental Health Protection Model for Disasters Risk Management (*Modelo de Protección de la Salud Mental en la Gestión del Riesgo de Desastres* [MPSMGRD]) [38] that reformulated the 2011 Manual for the Protection and Care of Mental Health in Emergency and Disaster situations [39] that pleaded for a “disaster culture” which placed considerable emphasis on the mental health consequences of disasters. The MPSMGRD is organised around seven principles (human rights and equity, *primum non nocere* (first, do no harm), prevention, participation, mutual aid, intersectorality, and stepped use of resources) and three approaches (social determinants of health, life course, and community-based action) [38].

Other significant social and natural events in Chile that have resulted in traumatic events include the civic-military dictatorship, characterized by serious human rights violations, which resulted in the death and disappearance of more than 3,000 people and more than 30,000 victims of political imprisonment and torture between 1973 and 1989 [40, 41]. More recently, Chile experienced considerable social unrest in 2019, months before the onset of COVID-19 pandemic, giving way to a potentially traumatic scenario, as also experienced in other countries [42].

In Argentina, taking into consideration the last 50 years, significant chronological milestones of trauma include: political violence during the 1970s that culminated with atrocious State violence during the military regime that ruled the country from 1976 to 1983 (with an estimated 30,000 deaths or missing persons), the Malvinas war in 1982 (650 soldiers dead), the Jewish AMIA bombing in 1994 (85 deaths), the “República Cromañón” nightclub fire in 2004 (194 young people dead), the 2012 train collision against the “Once” train station terminal (52 deaths) and, more recently, COVID-19 related deaths (130,000 deaths), with many more survivors and relatives of these victims (with the entire country affected by all these events). Other significant natural disasters in the last century included the 1944 earthquake in San Juan (killing almost 10% of the local population estimated at 10,000 deaths), and the 1973 San Justo tornado in the Province of Santa Fe (62 deaths). Other catastrophes, despite having a lower number of deaths, generated massive displacement of people and also became sources of trauma. Furthermore, PTEs derived from insecurity (e.g., being robbed or attacked) and affecting individuals, families, or small groups, are major concerns in the general population and also cause trauma.

Argentina is protected by the National System for the Integral Management of Risk [43], dependent on the Chief of Cabinet and the National Ministry of Security in conjunction with the 24 jurisdictions and multiple national entities (e.g., Environmental Ministry, Sustainable Development Ministry). The latest plan (2024–2030) includes training in emergency response and risk management [43], and incorporated a participatory process to make the necessary adaptations to the National Plan for the Reduction of Disaster Risks. The salient results of this participatory process were community suggestions with regards to *spreading catastrophe knowledge and awareness among the general population* and *training* [43].

In the face of the COVID-19 pandemic, a well-known specialist in catastrophes was named as chief director of the National Direction of Mental Health and Substance Abuse in 2021. Furthermore, an *Action Plan for the Building of a National Mental Health Response Network and Psychosocial Support for Emergencies and Disaster* was developed, aiming to provide Primary care and Secondary care workers with self-care tools and mental health care tools for the entire health care team in the frontline of response to COVID-19 emergency. WHO PFA was adopted as a standard for mental health care of individuals who have experienced a PTE. In addition, the Argentinian subsidiary of Red Cross also offers a 6-hour training course on PFA and psychosocial support specifically intended for lay people from the community [44].

Notwithstanding this notable progress in both Chile and Argentina, COVID-19 revealed significant gaps, increased incidence of psychological distress, anxiety, and depression [45, 46], and considerable room for improvement with regards to mental health support at a community level.

### Mental health first aid and potentially traumatic events

The Mental Health First Aid (MHFA) training courses were developed to equip community members with the needed skills to recognise when someone is developing a mental health problem (e.g., depression, drinking problems, and for someone who shows initial signs of trauma) and to assist them by providing mental health first aid until the crisis is resolved or further care is provided by the health care team [47]. Training is primarily intended for lay people from the community, and is not intended to replace professional training for first responders. MHFA training is not restricted to providing help for people who have experienced PTEs, but it includes content relating to trauma in its curriculum and is well suited to building capacity to respond to a broader range of mental health problems and crises in the longer term. Since the course was intended for the general community, there are no restrictions on admission to the course.

MHFA training is based on guidelines that were created using the Delphi expert consensus method and informed by the lived experience of people with mental health problems and those who care for them in addition to health care professional experts [48]. Studies were initially conducted in Australia and more recently in several non-English speaking countries such as China, Sri Lanka, Brazil, Chile, and Argentina.

The first guidelines to help individuals exposed to a PTE were developed by MHFA- Australia in 2008 [49, 50] and comprised specific items for adults and for children. The adult guidelines included 65 items and three major sections: actions that should be taken immediately after an event has occurred, assisting in the weeks following the traumatic event, and differentiating between people who are recovering normally and those who are in need of professional assistance [49]. This included: first priorities for helping someone after a traumatic event, priorities when helping after a mass traumatic event, how to talk to someone who has just experienced a traumatic event, how to help the person to cope over the next few weeks or months, and when the person should seek professional help. In 2019, these guidelines were updated and included a new section on how a first aider can assist a person after a disclosure of abuse. Addressing trauma in children was addressed in separate guidelines.

This study aimed to use the Delphi expert consensus methodology to culturally adapt guidelines for lay members of the community interested in providing mental health first aid to a person after a PTE in Chile and Argentina.

## Methods

Following previous studies conducted by our team and other adaptations for MHFA guidelines [51–53], we developed culturally adapted MHFA guidelines to train mental health first aiders supporting people exposed to a PTE in Chile and Argentina. We used a Delphi expert consensus approach, recruiting people with lived experience and mental health professionals to select the information to be included in the guidelines. The Delphi expert consensus encompassed four stages: [1] The development of the first-round survey; 2) Panel recruitment; 3) Data collection and analyses, and [4] Guidelines development.

The present study was part of an initiative to culturally adapt MHFA guidelines for Chile and Argentina on five key topics: depression, psychosis, alcohol, trauma, and suicide risk. The study received ethical approval from the University of Melbourne (in Australia), the University of Palermo (Argentina) and the University of Chile (Chile).

### Round 1 survey development

The first stage of the study was to develop the first-round questionnaire. We based our questionnaire on the first-round statements included in the 2019 Australian revision of the 2008 mental health first aid guidelines to support people exposed to a PTE [54]. Bilingual members of the team (MA and TT) translated the items to Spanish, culturally adapting the terms to ensure pertinence. Following this, the entire team decided on integrating or modifying items considering the countries’ context. A total of 158 items were incorporated for evaluation by the local experts in the first round. “First aider” was translated as “*Asistente de primeros auxilios*” or “*Asistente*”; “potentially traumatic event” was translated as “*evento potencialmente traumático*”. No additional items were suggested by the local research team at this point. One hundred and forty eight items were identical to Australian Round 1 items, three items were slightly modified for Chile and Argentina (but still comparable) and one item rated by the Chilean and Argentinian experts was the result of merging two separate items presented to the Australian experts (*The first aider should try to appear calm* AND *The first aider should try not to appear rushed or impatient* were transformed into *The first aider should be calm in the face of the trauma and try not to appear rushed or impatient*).

Statements were grouped into eight sections. See Table 1.

**Table 1:** Round 1 sections and examples of items

*Section 1: Background information (6 items)*
The first aider should be aware of the initial responses that are common following a potentially traumatic event.
The first aider should know what signs and symptoms can indicate there is a problem after a potentially traumatic event.
*Section 2: Actions to be taken immediately at the scene of a potentially traumatic event (29 items)*
The first aider should be calm in the face of the trauma and try not to appear rushed or impatient.
If the first aider feels that they are not emotionally capable of supporting the person, they should try to find someone else who is.
*Section 3: What to do at the site of a potentially traumatic event when professional helpers are already at the scene (5 items)*
The first aider should follow the directions of professional helpers at the scene
The first aider should not offer food or drink to the person without the permission of the professional helpers
*Section 4: Talking about the trauma (49 items)*
The first aider should encourage the person to talk about their feelings, but only if the person feels ready to do so
If the person repeatedly talks about the potentially traumatic event, the first aider should be willing to listen
*Section 5: Experiences of abuse (31 items)*
The first aider should be aware of any local mandatory reporting laws
If the first aider sees physical signs of abuse (for example, repeated bruising), they should discuss their concerns with the person.
*Section 6: Providing support during the following weeks and months after a potentially traumatic event (18 items)*
The first aider should discourage the person from making any major life decisions or big life changes, if at all possible.
The first aider should be aware of the type of professional help available to people who have experienced trauma
*Section 7: Encouraging professional help (10 items)*
The first aider should encourage the person to seek professional help if the post-trauma symptoms are interfering with their usual activities for 4 weeks or more
The first aider should encourage the person to seek professional help if they can’t stop thinking about the trauma for 4 weeks or more
*Section 8: Adolescents (10 items)*
The first aider should be aware of the ways in which an adolescent may respond differently to a potentially traumatic event compared to an adult.
The first aider should not hide information from the adolescent in an attempt to protect them.

### Recruitment of experts for both panels of experts

We invited participants for one of two panels: lived experience and healthcare professionals. The panel comprising experts with lived experience included participants with previous exposure to a traumatic event or because they self-identified as informal caregivers of people with trauma-related mental health issues (e.g., family or friends of a person who has PTSD). Panel two comprised health professional experts, including health providers as well as researchers and decision-makers. Both panels were recruited in Chile and Argentina. Participants were recruited by six members of the research team (MA, EL and SAG, Argentina; EE, IZ and TT, Chile).

We recruited the experts using a snowball strategy, starting with direct or referred contacts who had lived experience or were health professionals with experience working with people who had experienced trauma. Several participants from the lived experience panel were recruited from an association dedicated to offering support to persons who had been involved in the “*República Cromañón*” nightclub fire in 2004. The lived experience panel was also invited through digital posts on the participating universities’ social networks. Those interested in participating in the study registered on a Google form and were contacted via mail by both countries’ teams. They all received invitations explaining the study and the expectations that experts would help in developing guidelines on how to help someone who has experienced a potentially traumatic event. A broad definition of “person who has experienced a potentially traumatic event” was adopted without further specification. Participants declared their primary source of experience (as a health professional or as someone with lived experience) and also declared if they had an additional source of expertise. As in our previous study designs [55–57], experts invited to participate had to fulfil the following criteria:

- Lived experience expert panel, self-identified as having experienced distress following a potentially traumatic event or caring for a person with past experiences of trauma. Participants previously exposed to traumatic events were asked if they felt well enough to participate.
- More than four years of experience working as a healthcare professional with expertise in helping persons with traumatic experiences. Eligible types of professions included: psychologists, general practitioners, nurses, psychiatrists, social workers, and occupational therapists.
- Aged 18 years old and above.

### Data collection and analysis

Surveys for both rounds were conducted online through Qualtrics software. Data for the first round was collected between March 22, 2020, and March 28, 2022. The first round was severely affected by COVID-19 related restrictions which posed significant limitations to the distribution of the survey through health services in Chile and forced a considerable delay to finish data collection. Data for the second round was collected between July 7, 2022, and November 29, 2022.

The participants rated the importance of each statement to be included in the final mental health first aid guideline for individuals exposed to a PTE in Argentina and Chile. They evaluated the statements on a 5-point Likert scale (1 = essential, 2 = important, 3 = unsure, 4 = not important, 5 = should not be included). At the end of each subsection or after each 10 items (whichever came first), open-text response boxes were presented to allow participants to comment or suggest new recommendations that they felt were important to incorporate into the final guidelines. MA and TT elaborated new items based on these first-round suggestions. All these items were revised by NR before including them in the second round to ensure that they were actionable, applicable, and different from those already presented in the first round. Items in the first-round survey were immediately accepted if at least 80.0% in both panels rated them as “essential” or “important”. Moreover, items were re-rated in the second round if 70.0–79.9% of the experts in one of the panels and at least 70.0% of the other panel rated them as “essential” or “important”. Statements rated as “essential” or “important” by less than 70.0% of participants in one panel were immediately excluded. Additionally, items that did not receive at least 80% support but, according to the local experts, had the potential to be included in the final guidelines and had been the subject of suggestions related to language or need for clarification were reformulated and included in the second round for re-rating (*n* = 6). In Round 2, items with an acceptance rate of at least 80.0% by one panel and at least 75.0% by the other panel were selected for the final guideline.

We used Spearman correlations to evaluate the level of agreement between panels in each round.

The analysis was performed in IBM SPSS version 28.

### Guidelines development for Chile and Argentina

MA and TT consolidated the recommendations from the two rounds of surveys into a preliminary guideline document. The entire team reviewed the draft and provided input for a new version.

## Results

**Fig. 1:**
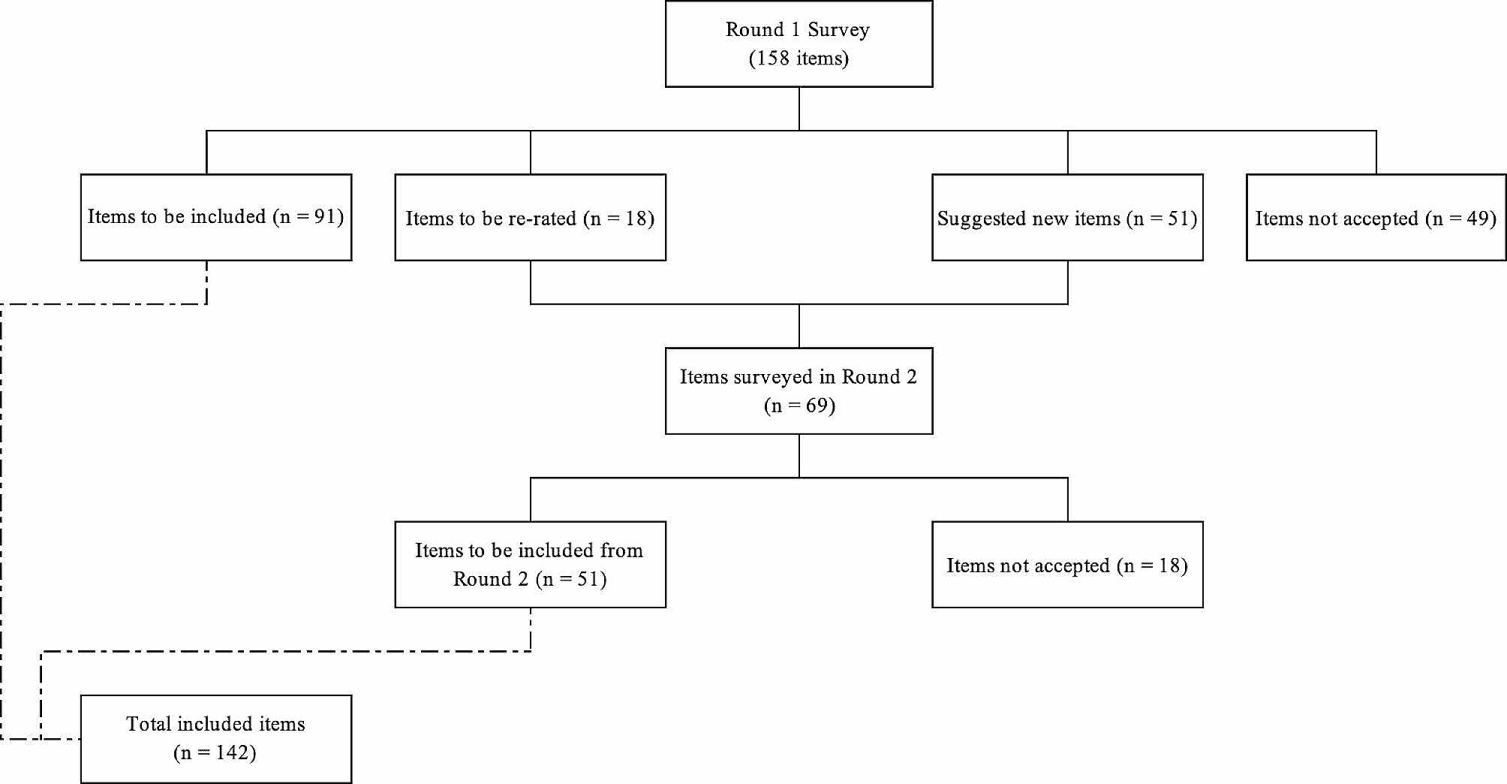
Overview of accepted and rejected items

Figure 1 shows the overall process of including the statements over the two rounds.

### Round 1

We received a total of 67 answers for the Round 1 questionnaire. The professional panel (*n* = 41) included more answers from Chile (*n* = 25) than Argentina (*n* = 16) and included 29 psychologists, five psychiatrists, three nurses, two occupational therapists, one social worker and one general practitioner. The mean number of years of experience as a health professional was 17.4 years, and 46% of the participants were between 35 and 44 years old. 77% were female (*n* = 31), 22% males (*n* = 9), and one participant self-identified as other gender. From those who identified themselves as professionals in their primary role, five of them were also consumers and one a caregiver.

The lived experience panel (*n* = 26) included more participants from Argentina (*n* = 16) than Chile (*n* = 10). Eighteen were consumers (69%) and eight were informal caregivers and/or relatives (31%). Of those who identified themselves as consumers in their primary role, six were also healthcare providers; and of those who identified themselves as caregivers in the primary role, one was also a healthcare worker. A total of 62% were females (*n* = 16) and 38% were males (*n* = 10). See Table 2 for a summary of the sociodemographic characteristics of participants.

**Table 2:** Sociodemographic characteristics of participants

	Round 1				Round 2			
	Argentina		Chile		Argentina		Chile	
	n	%	n	%	n	%	n	%
**Lived Experience**								
Gender								
Female	7	44.0	9	90.0	6	50.0	6	85.7
Male	9	56.0	1	10.0	6	50.0	1	14.3
Other	0	0.0	0	0.0	0	0.0	0	0.0
Total	16	100.0	10	100.0	12	100.0	7	100.0
**Type of LE**								
Consumers	9	56.0	9	90.0	7	58.3	7	100.0
Caregivers	7	44.0	1	10.0	5	41.7	0	0.0
Total	16	100.0	10	100.0	12	100.0	7	100.0
**Age**								
25-34	6	37.5	6	60.0	2	16.7	3	43.0
35-44	7	44.0	4	40.0	8	66.7	4	57.0
45-54	1	6.0	0	0.0	1	8.3	0	0.0
55-64	2	12.5	0	0.0	1	8.3	0	0.0
65+	0	0.0	0	0.0	0	0.0	0	0.0
Total	16	100.0	10	100.0	12	100.0	7	100.0
**Professionals**								
Gender								
Female	11	68.8	20	80.0	7	63.6	16	76.2
Male	5	31.3	4	16.0	4	36.4	4	19.0
Other	0	0.0	1	4.0	0	0.0	1	4.8
Total	16	100.0	25	100.0	11	100.0	21	100.0
**Age**								
25-34	0	0.0	7	28.0	0	0.0	2	9.5
35-44	4	25.0	15	60.0	3	27.3	15	71.4
45-54	4	25.0	2	8.0	3	27.3	3	14.3
55-64	4	25.0	0	0.0.0	3	27.3	0	0.0
65+	4	25.0	1	4.0	2	18.2	1	4.8
Total	16	100.0	25	100.0	11	100.0	21	100.0
**Profession**								
Psychologist	12	75.0	17	68.0	9	81.8	13	71.4
Psychiatrist	4	25.0	1	4.0	2	18.2	0	0.0
Nurse	0	0.0	3	12.0	0	0.0	3	14.3
General Practitioner	0	0.0	1	4.0	0	0.0	0	0.0
Other	0	0.0	3	12.0	0	0.0	3	14.3
Total	16	100.0	25	100.0	11	100.0	21	100.0

Out of the 158 items rated in the Round 1 survey, 91 items (57.6%) were endorsed as essential or important by 80% or more of the experts in both panels. Other 18 items (11.4%) were re-rated in Round 2, and 49 (31.0%) items were rejected (Fig. 1). Overall endorsement rates were 79.5% for the lived experience panel and 80.2% for the health professional panel, showing preliminary consistency.

### Round 2

A total of 51 answers were received in Round 2, including 32 from the healthcare professional panel (response rate of 78.0%) and 19 from the lived experience panel (response rate of 73.1%). No new participants were added in Round 2. The Round 2 questionnaire comprised 18 items from Round 1 to be re-rated and 51 new items suggested by the local experts (Fig. 1)^1^. Out of the 69 items rated in Round 2, 73.9% (*n* = 51) were endorsed by both panels and 26% (*n* = 18) were rejected. Six Round 2 items (8.7%) had a different formulation of statements not accepted during Round 1, including four items in the re-rate range and two with less than 70% endorsement in at least one panel. Despite having been rejected, experts’ comments indicated that a new formulation of those items would have been endorsed and were thus rephrased by the research team and tried in the second round. Two statements in the first category and one in the second category were finally endorsed.

### Similarities and differences between the spanish-language guidelines for Chile and Argentina and the English-language guidelines

Unlike previous studies to adapt other English MHFA guidelines to Chile and Argentina [55, 56], Round 1 statements were not comparable to the finalised English guidelines but to Round 1 statements evaluated by Australian experts in 2019. This situation offered us the opportunity to compare Australian with Chilean and Argentinian opinions towards a very similar set of statements. Australian panellists rated 171 items in Round 1 while the Chilean and Argentinian panellists rated 158. The 13-item difference included 9 statements regarding a two-week temporal window to encourage the person to seek mental health care (e.g., if the post-trauma symptoms are interfering with their usual activities, acting differently, etc.). Chile and Argentina only evaluated the four-week temporal window to encourage mental health consultation, while Australia evaluated both versions (two and four-weeks temporal window). An additional three items were not presented to the Chilean and Argentinian experts in either round due to a mistake during the adaptation process: *"The first aider should encourage the person to seek professional help if they misuse alcohol or other drugs to deal with the trauma at any time; If the first aider is concerned that the person is at risk of harm from someone else, they should encourage the person to call the police and report the situation; If the person’s loved ones or friends are not present, the first aider should offer to contact them"*. The remaining single item difference was the Australian items merged into one statement for Chile and Argentina.

Australian experts endorsed 53.2% of the items (*n* = 91) and rejected 33.9% (*n* = 58) out of a total of 171 statements evaluated during Round 1 (a remaining 12.9% was re-rated in a second round); Chilean and Argentinian experts endorsed 57.6% of the items (*n* = 91) and rejected 31.0% (*n* = 49) out of 158 statements evaluated during Round 1 (a remaining 11.4% was re-rated in Round 2). In total, Australian experts endorsed 56.3% of the statements (*n* = 103) over the whole rating process while Chilean and Argentinians endorsed 67.9% of the statements (*n* = 142); Australians rejected 43.7% of the statements (*n* = 80) while Chileans and Argentinians rejected 32.1% of the statements (*n* = 67). It is noteworthy that the Australian study used a more restrictive criteria for endorsement in Round 2 (i.e., at least 80% of both panels had to endorse the item as “essential” or “important” in order to incorporate it to the final guidelines). An additional four items would have been endorsed if Australia had used the same criteria of Chile and Argentina, and four items endorsed by the Chilean and Argentinian experts would have failed the cut-off line for inclusion in Australia (another nine items suggested during Round 1, and never tried in Australia, were also accepted for inclusion in Chile and Argentina but would have been rejected had been used the Australian–more restrictive–criteria for inclusion). There was a good level of agreement between the experts in both studies, with Chilean and Argentinian experts endorsing 81.6% of the same items endorsed by the Australians, but also endorsing 20.0% of the items not endorsed by the Australians. Interestingly, several original English statements were more likely to be accepted for inclusion in the final guidelines by the Chilean and Argentinian experts than by the Australians, including items relating to responses to a person in immediate danger or disclosure of abuse. In addition, the former experts suggested many more new items than the later (51 versus 13 statements).

The lived experience Chilean and Argentinian panellists rejected 12 statements endorsed in Australia, including the following: the first aider should prioritise their self-care over supporting others, when concerned that the person is at risk of harm from another person the first aider should do nothing that places themselves at risk, the first aider should admit that they lack information if this is the case, and not be afraid of admitting it if they do not know what to say).

Chilean and Argentinian experts endorsed 17 statements rejected in Australia, including two adolescent specific items (i.e., *If the adolescent has experienced a potentially traumatic event that is receiving media coverage, the first aider should try to limit their exposure to this media coverage*; *"If appropriate to the relationship, the first aider should contact the adolescent’s school about any additional support they may need*). Other statements included in the Chilean and Argentinian guidelines but not accepted in Australia included: *"If the person discloses abuse that happened in the past, the first aider should tell the person that they believe them*; *If the person talks repetitively about the potentially traumatic event, the first aider should listen*; *If the person wants to tell their whole story about the potentially traumatic event, the first aider should give the person enough time to do so*; *The first aider should discourage the person from making any impulsive decisions because they may not be thinking clearly*; *If the first aider does not know the person, they should find out the person’s name and use it when talking to them"*.

Out of the 51 new suggested items by the Argentinean and Chilean experts that were evaluated in in round 2, 78.4% (*n* = 40) were finally incorporated to the final guidelines, including: the first aider adapting their language to the characteristics of the person with regards to age, gender and/or cultural group; requesting professional help in the event of any physical or emotional emergency of the person who has experienced a traumatic event; considering asking for support when the person has special needs (e.g., reduced mobility, sensory disabilities, etc.); it being essential to interpret non-verbal language to understand when it is safe to approach, when it is safe to talk or when it is better to just be present; If the person discloses any abuse associated with criminal activity, the first aider should take into account how difficult it is to report an abuse, not put pressure on them and convey serenity, and provide support and ensure that the person is protected.

See supplementary file 1 for the final Spanish guidelines and file 2 for a full list of the statements endorsed and rejected in each round, plus the final list of items endorsed and the final list of items rejected across the two rounds.

### Similarities and differences between the lived experience and health professional panels

During Round 1, the level of agreement between panels was strong (Spearman *r* = 0.84). Only 5.1% of the items (*n* = 8) were endorsed by more than 80% of one of the panels and rejected (with less than 70% endorsement) by the other panel. Disagreements of 20% or more between panels were uncommon, accounting for only 13 statements. A considerable agreement (less than 10% difference between panels) comprised 65.9% of the items during Round 1 (*n* = 104), including five items with 100% agreement by both panels.

Local experts unanimously endorsed the following original English statements: *"The first aider should create a safe environment for the person, e.g. by moving away from traffic, fire or debris*; *The first aider should try to remain calm when talking with the person, regardless of the person’s emotional state*; *If the person discloses any abuse associated with criminal activity, the first aider should encourage the person to seek help from an appropriate support service*; *If the first aider is concerned that the person is at risk of harm from another person, they should help the person identify other people who can provide support*; *The first aider should be aware of the type of professional help available to people who have experienced trauma"*.

The Round 2 level of agreement between panels was less strong (Spearman *r* = 0.59). However, agreement included 8.7% of the evaluated items (*n* = 6) with absolute agreement by experts in both panels. These items, suggested by the panellists during Round 1, comprised several aspects pointing to the importance of recognising the limitations of the first aider’s role: *The first aider must know their limitations not only as a person but also in terms of the scope of their role*; *In the absence of professional helpers, if the first aider identifies the person’s needs that they cannot satisfy, they must evaluate the priorities and work with the available support services*; *If not sufficiently trained, the first aider should consider asking for support when the person has special needs (e.g., reduced mobility, sensory disabilities, etc.)*; *The first aider must request professional help in the event of any physical or emotional emergency of the person who has experienced a traumatic event*.

With regards to disagreements between panels, local experts had opposite perspectives about whether or not a person exposed to a PTE could recover without professional help. The professional panellists endorsed that “*The first aider must be aware that there are people who recover from a potentially traumatic event without the need for professional help”*, but only 47.4% of the lived experience panellists endorsed this statement. On the contrary, 89.5% of the lived experience experts agreed that “*The first aider must be aware that it is better for a person who has experienced a potentially traumatic situation to always have professional support in a preventive way”*, while only 65.6% in the professionals panel endorsed this statement.

Other statements with significant disagreement between panels–the lived experience panellists being less in favour than professionals– included: *"If the person wants more information about the event, the first aider should try to give them all the information they request unless it may cause further stress or impulsive behaviour that is detrimental to the person*; *The first aider should not offer religious comfort unless they are sure that the person shares these ideas*. In both cases, health professionals endorsed these items, but the lived experience experts rejected them".

In contrast, the lived experience panellists were more prone to accept that *If the person seems distressed, the first aider can offer to do a relaxation exercise together* (78.9% endorsed this statement in the lived experience panel), and *If the first aider is concerned that the person is at risk of harm from another person, they should offer to call an appropriate helpline on behalf of the person, e.g. family violence helpline* (84.6% endorsed the statement), but such strategies were endorsed by almost only half of the professional panellists.

## Discussion

The present study aimed to use the Delphi expert consensus method to adapt the Australian guidelines for community members wishing to provide mental health first aid to someone who experienced a PTE in Chile and Argentina. This was achieved by a two-round Delphi survey, involving mental health professionals and people with lived experience (either their own or as informal carers) and was designed to establish the actions on which both groups could agree. The English statements were mostly endorsed by the local experts and 102 items were incorporated from the original 158 items submitted for their evaluation. The final guidelines included 142 statements. Lived experience and healthcare professional panellists showed a high level of agreement on most of the rated items.

### Delineating the role of community members offering MHFA in Chile and Argentina

The panellists accepted the benefit of having lay persons from the community trained to provide psychological support to reduce the initial distress caused by a PTE until the crisis is resolved or further health care is provided by the healthcare team. From the original English statements evaluated by the local experts, an even larger number than those endorsed by the Australian experts were included in the final guidelines --pointing to a high level of acceptance of the first aider’s role. However, the local panellists suggested and accepted several items that trimmed down first aider’s scope in favour of mental health professionals. This was not only suggested by the members of the professional panel but also by the lived experience experts, who even recommended that the first aider be aware that it is better for a person who has experienced a PTE to always have professional support to assist with prevention.

Several statements alluding to considering the limitations of what first aiders should do received unanimous acceptance. According to the local panellists, the first aider should not only seek to collaborate with (and follow directives of) professional first responders if they are present but also know their limitations as a person and in terms of the scope of their role. A similar suggestion was seen in Brazil; in the context of adapting the 2009 Australian MHFA guidelines for trauma, the Brazilian team emphasised first aider’s need to recognize their own limits and respect them [19]. Interestingly, the Chilean and Argentinian lived experience panellists did not endorse first aider’s self-protection, possibly due to a view that protecting the victims is the most important thing (something that contradicts PFA principles that underscore the importance of self-care in a helping situation [35]) and suggests the lived experience panellists’ expectation of a first aider’s “heroism” [58]. Accordingly, they rejected items about the first aider’s need to prioritise their self-care over supporting others, and that when concerned that the person is at risk of harm from another person they should do nothing that places themselves at risk. Such consideration with regards to the first aider’s role aligns well with a COVID-19 study in Latin America that was named “HEROES” and aimed to understand how working during the pandemic had impacted healthcare professionals’ mental health [59]. Accepting the limitations of first aider’s role did not include prioritising their self-care. Notably, this consideration for disregarding self-care was absent in other local mental health first aid guidelines that adapted the Australian guidelines; when helping someone with drinking problems [55], depression [56], psychosis [57] or suicide risk [60], Chilean and Argentinian experts considered that the first aider’s self-care was indeed a priority.

Furthermore, similar to the other MHFA Delphi studies in Chile and Argentina, those for drinking problems [55], depression [56], psychosis [57] and suicide risk [60], the local experts were somewhat reluctant to expand the role of mental health first aiders when helping someone exposed to a PTE. Nevertheless, the panellists did endorse that first aiders show openness, listen empathetically and be willing to listen even when the person is being repetitive; they endorsed items about the first aider being able to interpret non-verbal language to understand when it is safe to approach, when it is safe to talk or when it is better to just be present. As much as possible, they should try to work as a team with other helpers (including other first aiders) and consider ways to provide support and accompany the network of people close to those who have lived through a traumatic experience. Helping not just the person but also family and significant others of individuals exposed to a PTE aligns well with the prevailing social ethos in many Latin American cultures, including *familism* (the cultivation and prioritisation of supportive family relationships) and the importance allotted to social bonds [61]. Providing mental health first aid was not recommended as a solitary task and those giving support were recommended to consider the networks of the person.

Lived experience panellists considered that first aiders should be able to offer reliable help and be adequately trained or, otherwise, not be part of the response team. Notwithstanding, both panels in Chile and Argentina accepted that “*If the person wants to talk about the potentially traumatic event but this is too distressing for the first aider, they should find someone else for the person to talk to”*. During a comparable study in China [62], this statement was not endorsed. In turn, while Chinese panellists endorsed that “*If the person starts a sensitive conversation and the first aider doesn’t think this is the ideal place to talk with the person, the first aider should suggest that they find an environment that is safe, comfortable, and free from distractions”*, this was not accepted by the lived experience panel in Chile and Argentina, as if local people with lived experience had valued not to interrupt the victim over looking for a safer and more comfortable place to talk with them. Future research to explore differences between the views of health professionals and people with lived experience of PTEs would be valuable.

### Incorporating mental health first aiders to the Chilean and the Argentinian response teams

In recognition of the relatively frequent situations in which the local population is exposed to PTEs (e.g., natural disasters, State violence, individual insecurity, human dependent catastrophes), Chile and Argentina have made considerable progress towards a timely and adequate response, paying particular attention to the mental health of individuals exposed to trauma and disasters. Both countries welcomed PFA in collaboration with WHO and Pan American Health Organisation (PAHO). In addition, community participation has been underscored by both countries as a way of increasing responsiveness [38, 43]. Despite the potential benefit of having more people in the community knowledgeable about mental health and available to provide psychosocial support --a well-known strategy to buffer the consequences of PTEs– and its perfect alignment with the locally claimed community participation, widespread dissemination of the MHFA training based on the guidelines requires further exploration of issues relating to local laws as well as supervision, monitoring and funding for rollout. Other difficulties envisioned by the local panellists will also need further consideration. Firstly, making the necessary adjustments of a novel role for lay persons when it is finally put into practice will require some system changes. Secondly, reconciling contradictory expectations, including the awareness of limitations with the prioritisation of the helping role and scant room for self-care, based on a ‘heroic’ idea [63], will need careful consideration to avoid vicarious trauma and burnout. Last, but not least, achieving the acceptance of professional first responders to work as a team with the first aiders calls for persuasion and political will.

### Strengths and limitations

A significant strength of this study comes with the research design that gives equal weight to the views of health professionals and people with lived experience. This is of utmost importance considering that the objective was to culturally adapt the recommendations for lay members of the community, which is unlikely to be achieved only with input from professionals. Moreover, community participation has been considered crucial in adequately responding to catastrophic events.

The higher number of endorsed items, and the large number of others newly suggested, points to the appropriateness of using the original list of English statements but also to the value of the cultural adaptation process.

Limitations included the absence of information on the types of traumatic events experienced by the lived experience panel, as well as the health-related consequences. Almost 23% of the participants dropped out after the first round and, additionally, they were not equally distributed by country; Chile contributed more experts in the professional panel than Argentina, while contributing fewer experts in the lived experience panel. However, having participants from both Chile and Argentina supports the case for generalisability of the findings to other Latin American Spanish-speaking countries. We expect that further studies while implementing the guidelines will contribute to additional adaptations that could increase their relatability and potential for domestic use.

## Conclusion

A Delphi expert consensus study involving people with lived experience (either their own or as informal caregivers) and healthcare professionals was used to adapt for Chile and Argentina the mental health first aid guidelines for individuals who experienced a PTE. The adapted guidelines confirmed essential aspects of the original statements rated in Australia in 2019 and included several new aspects with regards to actions to be taken immediately at the scene of a PTE and in the weeks or months after the event, enlarging the need to accept the limitations of being a non-professional first aider while placing less emphasis on the importance of self-care. Further research on dissemination, acceptance, training, and usage of the guidelines in Argentina and Chile is still pending.

Supplementary information accompanies this paper.

1. Expert consensus Spanish guidelines for helping a person who has experienced a potentially traumatic event.
2. Statements that were presented to the panels and their ratings across 2 rounds of the study.

### Electronic supplementary material

Below is the link to the electronic supplementary material.

**Supplementary Material 1:** Expert consensus Spanish guidelines for helping a person who experienced a potentially traumatic event

**Supplementary Material 2:** Statements that were presented to the panels and their ratings across 2 rounds of the study

## Data Availability

The data supporting our findings is attached as Supplementary file 1, which contains all the statements that were presented to the panels and their endorsement rates across the two survey rounds.

## Footnotes

1 Two items would have required re-rating but were mistakenly not presented to the experts in Round 2. We used Round 2 criteria for Round 1 answers to these two statements and one of them was included in the final Guidelines (92.3% and 78.0% endorsement rate) and the other one was rejected (76.9% and 73.2% endorsement rate).

## Abbreviations

MHFA: Mental health First Aid
PFA: Psychological First Aid
PTE: Potentially traumatic event
PTSD: Post-traumatic stress disorder
