# Supplementary material for: Development of mental health first-aid guidelines for a person after a potentially traumatic event: A Delphi expert consensus study in Argentina and Chile
Source: BMC Psychiatry. 2024 Apr 17;24:291. doi: 10.1186/s12888-024-05631-4 (PMC11025165; doi:10.1186/s12888-024-05631-4)
Supplement: Supplementary file 1 — Supplementary Material 1: Expert consensus Spanish guidelines for helping a person who experienced a potentially traumatic event [file 12888_2024_5631_MOESM1_ESM.pdf]

MENTAL HEALTH FIRST AID
Chile - Argentina

CÓMO BRINDAR AYUDA A UNA PERSONA QUE HA EXPERIMENTADO UN EVENTO TRAUMÁTICO

GUÍA DE PRIMEROS AUXILIOS EN SALUD MENTAL

En esta guía encontrarás un conjunto de recomendaciones para ayudar a alguien que se encuentra atravesando una situación traumática (o, potencialmente traumática) o que pueda haberla experimentado en el pasado reciente. Estas recomendaciones están divididas en diferentes secciones: Información inicial que deberías tener respecto de las situaciones traumáticas y las formas de reaccionar a éstas; Acciones a tomar en el sitio mismo donde está teniendo lugar una situación que podría ser potencialmente traumática; Qué hacer en el sitio de una situación potencialmente traumática cuando ya hay asistentes profesionales en el lugar; Cómo hablar sobre el trauma (la experiencia, los sentimientos, los desafíos durante la conversación, la presencia de flashbacks); Cómo ayudar a alguien que experimentó o continúa experimentando situaciones de abuso; Cómo proporcionar ayuda en las semanas o meses posteriores a un evento potencialmente traumático; Cómo reconocer y sugerir la consulta con profesionales de la salud para abordar los efectos de una situación traumática; Particularidades de la ayuda a adolescentes.

Al finalizar podrás ver detalles de cómo se realizó esta guía y sugerencias para su utilización o consultas adicionales.

Qué es un evento potencialmente traumático

Un evento potencialmente traumático es una experiencia significativa y angustiante que pone en peligro la vida o representa una amenaza para el bienestar físico o psicológico de una persona. Muchos eventos, pasados y presentes, tienen el potencial de ser traumáticos, pero no todos los eventos comúnmente percibidos como traumáticos causarán una angustia extrema a una persona. Contrariamente, algunos eventos que pueden no ser traumáticos para algunas personas terminan siéndolo para otras.

Algunos ejemplos comunes de eventos que tienen el potencial de generar un trauma son: la violencia interpersonal (incluida la violencia familiar, el abuso infantil, el abuso de ancianos, la agresión física o sexual, el acoso, el asalto o el robo), los accidentes (como accidentes de tránsito o laborales), o presenciar algo violento para una tercera persona. Algunos eventos traumáticos son de carácter masivo y afectan de modo simultáneo a muchas personas, como por ejemplo la guerra, ataques terroristas, catástrofes durante eventos masivos, accidentes masivos, o eventos climáticos severos (como inundaciones, terremotos, huracanes, tsunamis, aludes, o incendios). Una tercera categoría de evento traumático lo configuran distintas formas de violencia institucional y de Estado, algo que ha tenido enorme difusión en América Latina y que aún sigue teniendo vigencia.

MENTAL HEALTH FIRST AID
Chile - Argentina

CÓMO BRINDAR AYUDA A UNA PERSONA QUE HA EXPERIMENTADO UN EVENTO TRAUMÁTICO

GUÍA DE PRIMEROS AUXILIOS EN SALUD MENTAL

¿Cómo reconocer las respuestas iniciales a una situación potencialmente traumática?

Es común que luego de experimentar un evento de estrés agudo que puede ser potencialmente traumático, las personas tengan respuestas psicológicas y conductuales que les generen malestar. Dentro de las respuestas podemos encontrar ansiedad, tristeza, enojo, aislamiento social, entre otras.

Estas respuestas son esperables y se contempla que disminuyan luego de algunos días desde la experiencia de estrés. Ahora bien, las personas pueden mantenerse experimentando respuestas negativas relacionadas con el evento. Las respuestas negativas al trauma más severas son el trastorno de estrés postraumático y el trastorno de estrés postraumático complejo.

La persona que ha experimentado un evento potencialmente traumático (de estrés agudo) puede presentar:

- Respuestas fisiológicas de ansiedad como hiperactividad (persona se mueve constantemente), sudoración y taquicardia (aumento del ritmo cardiaco);

- Respuestas cognitivas de ansiedad como pensamientos recurrentes, dificultad para concentrarse, y sensación de intranquilidad;

- Problemas de sueño (no poder quedarse dormido, despertar durante la noche o despertar muy temprano;

- Tristeza;

- Rabia o enojo;

- Inactividad;

- Aislamiento social;

- Estupor (la persona parece en estado de shock);

- Miedo o temor.

CÓMO BRINDAR AYUDA A UNA PERSONA QUE HA EXPERIMENTADO UN EVENTO TRAUMÁTICO

GUÍA DE PRIMEROS AUXILIOS EN SALUD MENTAL

MENTAL HEALTH FIRST AID
Chile - Argentina

Cómo reconocer el trastorno de estrés postraumático (TEPT)

La persona experimenta los siguientes síntomas durante al menos varias semanas y causan un deterioro significativo en el funcionamiento en áreas importantes (familiar, laboral, etc):

1) Volver a experimentar el evento o eventos traumáticos en el presente en forma de vívidos recuerdos intrusivos, flashbacks o pesadillas. La re-vivencia puede ocurrir a través de una o múltiples modalidades sensoriales y típicamente va acompañada de emociones fuertes o abrumadoras, particularmente miedo u horror, y fuertes sensaciones físicas;

2) Evitar pensamientos y recuerdos del evento o eventos, o evitar actividades, situaciones o personas que recuerden el/los evento/s;

3) Percepciones persistentes de una amenaza actual acentuada, por ejemplo, como lo indica la hipervigilancia o una reacción de sobresalto aumentada ante estímulos como ruidos inesperados.

El trastorno de estrés postraumático complejo (TEPT complejo) se caracteriza porque se cumplen con mayor gravedad todos los criterios de diagnóstico del trastorno de estrés postraumático además de los siguientes síntomas:

1) Problemas para regular sus emociones;

2) Creencias respecto de sentirse uno mismo disminuido, derrotado o sin valor, así como sentimientos de vergüenza, culpa o fracaso relacionados con el evento traumático;

3) Dificultades para mantener relaciones y sentirse cerca de los demás.

Información inicial que deberías recordar

Es importante que seas consciente de las respuestas iniciales que una persona puede tener después de un evento potencialmente traumático, así como también los signos y síntomas que pueden indicar que hay un problema derivado de tal evento, y el rango de síntomas que pueden ocurrir a largo plazo.

Si estás en contacto con una persona que ha experimentado un evento potencialmente traumático debes tener presente que las personas pueden reaccionar de manera muy diferente y no debes esperar ninguna reacción en particular. Además, es importante que consideres que existen diferencias culturales en la forma en que las personas responden a un evento potencialmente traumático (por ejemplo, en algunas culturas, expresar vulnerabilidad o dolor en frente de extraños no se considera apropiado). Es importante que consideres que entre la gama de reacciones a un evento traumático que pueden existir la persona puede experimentar la culpa del sobreviviente, es decir, la sensación de que es injusto que otros hubieran muerto o resultaron heridos, mientras ella resultó ilesa.

Ten presente que algunas respuestas pueden parecer reacciones emocionales, pero son consecuencias de un síntoma físico (Ej. traumatismo encefalocraneano (TEC)) y eso requiere cuidados adicionales y consultas pertinentes.

Contacto inicial

Determinar si es seguro

Cuando recién interactúas con una persona que ha vivido un evento potencialmente traumático o de estrés agudo (sea alguien que conozcas o no), debes primero determinar si es seguro acercarse a la persona antes de tomar cualquier acción (por ejemplo, peligro de incendio, armas, etc.).

Crear un ambiente seguro

Debes intentar crear un ambiente seguro para la persona. Las siguientes son cosas que puedes hacer para crear un ambiente seguro para la persona:

- Alejarse de espacios que pueden ser potencialmente dañinos como, por ejemplo, alejándose del tráfico, incendio o escombros;

- Tratar de parecer tranquilo, sin dar señales de estar apresurado o impaciente;

- Si no conoces a la persona, intenta averiguar su nombre para darle seguridad y tranquilidad cuando hablas con ella y explicarle la función que cumples y por qué estás ahí.

Observar el estado físico y mental de la persona

Recuerda que es importante observar las señales de que el estado físico o mental de la persona esté disminuyendo, y estar preparado para buscar asistencia médica de emergencia para ella (por ejemplo, una persona aparentemente ilesa puede tener lesiones internas que tarden en hacerse evidentes, o una persona puede desorientarse repentinamente). Antes de actuar, debes observar qué otra ayuda ya se encuentra recibiendo la persona y si se necesita ayuda adicional. Si la persona ya está recibiendo ayuda, debes colaborar con los ayudantes y preguntarles si necesitan ayuda o un descanso. Si se necesita ayuda adicional debes obtener ayuda médica para la persona si es necesario y ponerte en contacto con la ayuda de emergencia adecuada (por ejemplo, ambulancia, policía, bomberos).

Determinar sus necesidades

Debes averiguar cuáles son las necesidades inmediatas de la persona (por ejemplo, alimentos, ropa, refugio, ayuda médica o apoyo emocional) y tratar de satisfacerlas; es importante que seas consciente de la comodidad y de la dignidad de la persona, y responder de manera acorde (por ejemplo. ofreciéndole a la persona algo para cubrirse y pidiéndole a los espectadores y a los medios que se vayan). Trata de minimizar la exposición de la persona a visiones y sonidos potencialmente molestos, por ejemplo, personas heridas o luces intermitentes. Ante la ausencia de ayudantes profesionales, si identificas necesidades de la persona que no puedes satisfacer, debes evaluar las prioridades y articular con los apoyos disponibles.

Atención a tu propio estado emocional

Antes de ayudar a la persona, debes considerar si te encuentras en un estado emocional adecuado para brindar apoyo a los demás. Si no estás emocionalmente en condiciones para apoyar a la persona, debes tratar de encontrar a alguien más que lo esté.

Cuanta información entregar

Si la persona solicita información, debes averiguar con los ayudantes profesionales qué información se le permite transmitir a la persona. Debes ser cuidadoso y procurar que la información que entregues sea pertinente, oportuna y veraz. Te debes capacitar en cómo y cuándo entregar información sobre un evento traumático.

Puede ser que otros estén involucrados en la información que te solicitan. En tal caso, debes procurar proteger la privacidad y dignidad de otros (terceros) al momento de entregar la información que la persona solicita. No debe hacer promesas que no puedas cumplir (por ejemplo, decirle “te llevaré a casa pronto”).

Si la persona parece abrumada o indecisa, debes ayudarla a tomar las decisiones necesarias (por ejemplo, para garantizar su seguridad). Debes desalentar a la persona de tomar cualquier decisión impulsiva porque puede que no estén pensando con claridad en ese primer momento.

Si consideras que alguien no está actuando con la única intención de ayudar a la persona (por ejemplo, tratando de obtener una entrevista en los medios o impidiendo que se reporte a las autoridades), debes tratar de proteger a la persona de esta situación.

Debes considerar que, en caso de situaciones traumáticas relacionadas con delitos, se te puede solicitar declarar como testigo y/o entregar tus datos de contacto.

Ayuda profesional en el lugar

Si ya existe ayuda profesional, debes seguir las instrucciones de los ayudantes profesionales en la escena y no debes criticar frente a la persona los esfuerzos que éstos hacen. Debes solicitar ayuda profesional ante cualquier urgencia física o emocional de la persona que ha experimentado un evento traumático. Si no estás capacitado, debes considerar pedir apoyo cuando las personas asistidas tengan necesidades especiales (ej. movilidad reducida, discapacidad sensorial, etc). Como parte de tu capacitación debes conocer los recursos y/o apoyos disponibles que se puedan necesitar en situaciones de catástrofe/ traumáticas.

Debes conocer técnicas de primeros auxilios básicos, maniobras de reanimación y poder determinar cuándo se requiere de ayuda médica de urgencia. Debes ser consciente que puede ser requerida tu capacidad física para contener

a la persona durante una crisis. Debieras estar atento a indicadores de riesgo de suicidio y conocer cómo brindar los primeros auxilios a personas en esa situación (por ej., conocer la Guía de primeros auxilios para riesgo de suicidio de Mental Health First Aid y su versión local de Primeros Auxilios en Salud Mental para Chile y Argentina)

En la medida que sea posible, debes intentar trabajar en equipo con otros asistentes.

Hablando con la persona acerca del trauma

Cuando una persona ha experimentado un evento de estrés agudo que es potencialmente traumático no debes decirle a la persona cómo debe sentirse. Debes comunicarte con la persona como un igual, en lugar de como un experto superior. Debes tratar de mantener la calma cuando hablas con la persona, independientemente del estado emocional de ésta.

Utiliza un lenguaje claro y acorde a las características de la persona asistida según edad, género y/o grupo cultural. Debes considerar preguntar a la persona cómo desea que la llamen o cuáles son los pronombres con que se identifica. Procura ser empático, mostrar apertura y disposición, y tener escucha activa cuando interactúas con la persona. Evita hacer juicios de valor respecto del relato de la persona asistida.

Es esencial que puedas interpretar el lenguaje no verbal para entender cuándo es prudente acercarte, cuándo es prudente hablar o cuándo es mejor solo estar presente. Deberías prestar atención a tu postura física para asegurar un espacio de mayor confianza con la persona.

Debes intentar normalizar las emociones de la persona y asegurarle que sus reacciones son esperables dadas las circunstancias. Debes mostrar a esa persona que estás escuchando pacientemente, incluso cuando la persona no se esté expresando bien (por ejemplo, repitiéndose, hablando despacio o sin claridad).

Debes ser consciente de que la persona puede no recordar todos los detalles de un evento potencialmente traumático. Por otra parte, no debes obligar a la persona a hablar sobre lo sucedido o sobre sus sentimientos al respecto. Asimismo, si la persona habla repetitivamente sobre lo sucedido, debes escucharla. Si la persona expresa lo que siente o algo sobre el evento, no debes interrumpir a la persona para compartir sus propios sentimientos y experiencias. Si quiere contar su historia completa sobre el evento, debes darle a la persona el tiempo suficiente para hacerlo. Si la persona comienza a llorar, o parece estar tratando de no llorar, debes decirle a la persona que está bien llorar o expresar cualquier sentimiento que esté experimentando. Evita decir cosas que desanimen a la persona a expresar sus sentimientos (por ejemplo, “No llores” o “cálmate”). No debes comparar la experiencia traumática de la persona con la experiencia de otra persona.

Si la persona está reticente a confiar en ti, debes ser paciente y preguntar a la persona si aun así puedes ayudarla de alguna manera. Si la persona no desea hablar, debes considerar acompañarla en silencio o aguardar a cierta distancia por si requiere ayuda.

No debes emitir opinión sobre las responsabilidades de lo sucedido en el evento traumático. Especialmente decir nada que implique que lo que sucedió fue culpa de la persona. Tampoco debes decir nada que implique que la persona debería haber reaccionado o hecho algo diferente en el momento del evento potencialmente traumático, ni minimizar la experiencia de la persona (por ejemplo, “Podría haber sido peor” o “Estarás bien” o “Ya deberías haber superado eso”).

Si la persona no desea hablar contigo sobre cómo se siente, deberías ofrecer otras opciones (como llamar a una línea de ayuda o utilizar otros recursos de la comunidad).

Si la persona se siente avergonzada o culpable por la forma en que reaccionó, debes asegurarle a la persona que la gente no elige conscientemente cómo responder en estas situaciones, ya que estas reacciones suelen ser automáticas e instintivas.

Si la persona quiere hablar sobre el evento potencialmente traumático, pero esto te resulta demasiado angustiante, debes encontrar a alguien más con quien la persona pueda hablar.

Debes saber que el comportamiento, como la retracción, la irritabilidad y el mal humor, pueden ser una respuesta a lo sucedido, y debes tratar de no tomarlo personalmente. Trata de ser amable, incluso si encuentras que el comportamiento de la persona es desafiante.

Si la persona parece estar angustiada por la conversación, pregunta directamente si desea continuar conversando. Si la persona parece estar “atontada”, “ensimismada” o está

teniendo dificultades para comunicarse, no asumas que la persona no quiere hablar, pregunta a la persona qué necesita en ese momento, y no obligues a la persona a hablar sobre lo que la está angustiando. Puedes, también, ofrecer hablar en otro momento.

Qué hacer frente un flashback

Si la persona experimenta un flashback, ten en cuenta que los flashbacks suelen durar pocos minutos y debes esperar hasta que termine para hablar con la persona. Una vez que terminen, recuerda a la persona que está a salvo, llama su atención sobre el aquí y el ahora, por ejemplo, haciendo referencia al entorno inmediato. Evita movimientos bruscos o cualquier cosa que pueda asustarla, considera evitar el contacto físico y hacerlo solo luego de evaluar que eso será beneficioso para la persona en ese momento. Informa a la persona que existen formas de ayuda para manejar y superar los flashbacks y vivencias de desrealización.

Intenta decirle a la persona que los flashbacks y la vivencia de desrealización son reacciones esperables para alguien que ha experimentado un trauma. Procura ser empático, mostrar apertura y disposición, y tener escucha activa cuando interactúas con la persona.

Experiencias de abuso

Si la persona revela algún abuso asociado con una actividad criminal debes alentar a la persona a buscar ayuda de un servicio de apoyo adecuado y estar al tanto de cualquier ley local de notificación obligatoria. Debes tener en cuenta lo difícil que es denunciar un abuso, no presionarla, transmitirle serenidad, brindar apoyo y procurar que la persona esté protegida. En el caso de que un abuso esté ocurriendo, debes activar todos los dispositivos de ayuda para proteger a la persona en esa situación.

Debes ser consciente de que la persona que ha sufrido abuso puede no confiar fácilmente y puede ser reacia a buscar apoyo de otros. Si la persona revela un abuso, debes escucharla y saber que no tienes que proporcionarle soluciones o consejos.

Si expresas tus inquietudes sobre los signos de abuso físico y la persona lo rechaza, ten en cuenta que no significa necesariamente que tus preocupaciones estén fuera de lugar. Si la persona desestima tus preocupaciones, y ves posibles signos de abuso en el futuro, debes volver a plantear tus preocupaciones con la persona. Debes considerar que una persona que es víctima de abuso doméstico es posible que minimice y/u omita información para evitar riesgos.

Si estás preocupado de que la persona corre el riesgo de sufrir daños por parte de otra persona, debes trabajar con la persona para identificar qué pasos tomar a continuación para mantenerla segura, ofrecerle opciones para mantenerla segura, por ejemplo, ayudándola a encontrar alojamiento alternativo. En estos casos, deberías: ayudar a la persona a identificar a otras personas que puedan brindar apoyo, ofrecer llamar a la policía y reportar la situación, alentar a la persona a llamar a una línea de ayuda adecuada (por ejemplo, la línea de ayuda para situaciones de violencia doméstica), ofrecer llamar a una línea de ayuda apropiada en nombre de la persona (por ejemplo, la línea de ayuda para situaciones de violencia doméstica).

Si la persona te pide que no le digas a nadie sobre el abuso que ha experimentado, debes respetar sus deseos, a menos que esté en riesgo de daño inmediato o a menos que el abuso constituya un crimen que sea forzoso denunciar. Siempre debes considerar las posibles repercusiones para la persona (por ejemplo, su seguridad o relaciones).

Si la persona revela un abuso que sucedió en el pasado debes hacerle saber a la persona que le crees, no expresar ningún sentimiento de incredulidad y agradecer a la persona por habértelo confiado.

Si la persona comienza a relatar detalles del abuso y es angustiante para ti, debes protegerte y alentar a la persona a hablar con un servicio de soporte en vez de contigo.

Facilitar a la persona información respecto de los medios institucionales a los que puede recurrir para salir de la situación de abuso (por ejemplo, lugares donde denunciar, pedir protección, centros especializados, etc.).

Cuida cómo y con quién compartes información para evitar la revictimización o estigmatización de la persona. Conoce tus limitaciones no solo como persona sino en lo referido a los alcances de tu rol. En líneas generales, debes ser consciente que apoyar a la persona puede producir desgaste o agotamiento y que, por tanto, cuando esto ocurra debes buscar apoyo y/o manejar otras estrategias para cuidar de ti mismo.

MENTAL HEALTH FIRST AID
Chile - Argentina

CÓMO BRINDAR AYUDA A UNA PERSONA QUE HA EXPERIMENTADO UN EVENTO TRAUMÁTICO

GUÍA DE PRIMEROS AUXILIOS EN SALUD MENTAL

Proporcionar apoyo en las semanas y meses posteriores a un evento potencialmente traumático

Incluso semanas o meses después de un evento potencialmente traumático, la persona puede tener días buenos y días malos. No hay un cronograma para lo que se puede esperar. Debes decirle a la persona que todos elaboran este tipo de situaciones a su propio ritmo y que es posible recuperarse de eventos traumáticos.

La persona puede ser más sensible a los eventos o al estrés pese a que a otros estos eventos puedan parecerles menores. Hay muchas cosas que pueden hacer recordar el trauma a la persona y causarle angustia. La persona puede recordar repentina o inesperadamente los detalles del evento y puede necesitar asistencia adicional en ese momento.

Los aniversarios de estos eventos y los informes de los medios de comunicación de experiencias similares pueden hacerle acordar del trauma a la persona y ella puede necesitar, entonces, apoyo adicional. Alienta a la persona a usar apoyos que sean específicos para el tipo de evento traumático que atravesó.

Debes ser consciente del tipo de ayuda profesional disponible para las personas que han experimentado un trauma así como alentar a la persona a buscar ayuda de un profesional que trata a personas que han sufrido un trauma. Puede ser beneficiosa la elaboración grupal en el caso de un evento traumático que involucre a muchas personas a la vez. Debes mencionar a la persona que no tiene por qué relatar el hecho traumático a todos quienes se lo soliciten.

Debes considerar, también, formas de prestar apoyo y acompañar a la red de personas cercanas de quien ha vivido una experiencia traumática.

¿Cuándo debe la persona buscar ayuda profesional?

Debes alentar a la persona a buscar ayuda profesional si después de 4 semanas o más le sucede que:

- Los síntomas postraumáticos interfieren con sus actividades habituales;

- Se siente muy molesta o temerosa;

- Los sentimientos de angustia siguen siendo intensos;

- Si actúa de manera muy diferente a como se solía comportar después del evento potencialmente traumático;

- Si sus relaciones importantes se ven afectadas como resultado del trauma (por ejemplo, si evita a su familia o amigos);

- Si se siente nerviosa o tiene pesadillas debido a o sobre el trauma;

- Si no puede dejar de pensar en el trauma;

- Si no puede disfrutar de la vida en absoluto como resultado del trauma;

- Si la persona abusa del alcohol u otras drogas para lidiar con el trauma.

Adolescentes

Debes ser consciente de las formas en que un adolescente puede responder a un evento potencialmente traumático y que éstas pueden ser diferentes en comparación con un adulto. Debes recurrir a un organismo de protección de derechos de niños, niñas y adolescentes si el adolescente continúa en una situación de peligro.

Si no tienes un vínculo preexistente con el adolescente, debes preguntarle si tiene un adulto de confianza que pueda apoyarlo. Si el adolescente no tiene un adulto confiable y apropiado con quien hablar, debes conectarlo con un servicio apropiado (por ejemplo, un programa comunitario, centro de salud o línea de ayuda).

Si es apropiado para la relación, debes comunicarte con la escuela del adolescente por cualquier apoyo adicional que pueda necesitar.

Si el adolescente ha experimentado un evento potencialmente traumático que está recibiendo cobertura de los medios, debes tratar de limitar su exposición a esta cobertura de los medios.

MENTAL HEALTH FIRST AID Chile - Argentina

CÓMO BRINDAR AYUDA A UNA PERSONA QUE HA EXPERIMENTADO UN EVENTO TRAUMÁTICO

GUÍA DE PRIMEROS AUXILIOS EN SALUD MENTAL

Si el adolescente no quiere hablar sobre lo que ha ocurrido, debes informar al adolescente que estás listo para hablar si él quiere, preguntarle si hay alguien más con quien preferiría hablar, y ayudarle a expresar sus sentimientos en lugar de hablar sobre lo que ha ocurrido. Debes preguntar cuáles son las mejores formas de comunicación que puede tener con sus pares y no realizar ninguna valoración sobre los modos de comunicación que el adolescente prefiera en ese momento.

Cómo se desarrollaron estas guías

Estas guías son el resultado de la consulta con expertos de Chile y de Argentina, incluyendo a profesionales de la salud y personas con experiencia vivida (propia o como cuidadores informales) que realizaron sus recomendaciones a partir de las guías originales en inglés desarrolladas por expertos de Alemania, Australia, Bélgica, Canadá, Estados Unidos de América, Francia, Irlanda, Noruega, Reino Unido y Suecia.

Los detalles de la metodología empleada por el equipo local pueden consultarse en la publicación: Agrest M, Tapia-Muñoz T, Encina E, Ardila-Gómez S, Alvarado R, Leiderman E, Reavley N. Development of mental health first aid guidelines for persons who have experienced a potentially traumatic event: a Delphi expert consensus study in Argentina and Chile. (en proceso de publicación).

Cómo utilizar estas guías

Esta guía reúne un conjunto de orientaciones generales para personas que han experimentado situaciones traumáticas o que se presume que pueden haber sido traumáticas. Sin embargo, cada persona es única y es importante que el apoyo que se le brinde esté adaptado a sus necesidades. En este sentido, puede que haya personas que no sean ayudadas aun siguiendo estas recomendaciones o que estas no sean apropiadas para algunas personas, aun cuando están formuladas en consideración a aspectos culturales de la población chilena y argentina.

Estas guías fueron desarrolladas por la Universidad de Palermo, la Universidad de Chile y la Universidad de Melbourne y están protegidas por el derecho de autor. Sin embargo, se pueden usar libremente si no es con fines de lucro.

Se recomienda citar como: Primeros Auxilios en Salud Mental, América Latina. Brindando ayuda a una persona que ha experimentado una situación traumática: Guía de primeros auxilios en salud mental. Universidad de Chile, Universidad de Palermo y Universidad de Melbourne, 2023.

Si tiene dudas respecto de su utilización puede dirigirse a:

Martín Agrest, magrest66@gmail.com
Thamara Tapia, thammytapia@gmail.com
Esteban Encina, esteban2@uchile.cl
Nicola Reavley, nreavley@unimelb.edu.au. +61 3 9035 7628
